# No difference in sexual behavior of adolescent girls following Human Papilloma Virus vaccination: a case study two districts in Uganda; Nakasongola and Luwero

**DOI:** 10.1186/1471-2458-14-155

**Published:** 2014-02-12

**Authors:** Judith Caroline Aujo, Sabrina Bakeera-Kitaka, Sarah Kiguli, Florence Mirembe

**Affiliations:** 1 Department of Paediatrics and Child health, Mulago National Referral Hospital, Kampala, Uganda; 2 Department of Paediatrics and Child health, Makerere University College of Health Sciences, Kampala, Uganda; 3 Department of Obstetrics and Gynaecology, Makerere University College of Health Sciences, Kampala, Uganda

## Abstract

**Background:**

Vaccination against Human Papilloma Virus (HPV) before sexual debut has been recommended by WHO as a primary prevention strategy against cervical cancer. In Uganda, vaccination against HPV started as a demonstration project among young girls in Nakasongola; and Ibanda districts. Studies have suggested that vaccination against HPV could result in risky sexual behavior and increase the risk of early sexual debut.

This study was done to compare the sexual behavior of HPV vaccinated and non vaccinated adolescent girls in two neighboring districts in Uganda; and to assess whether HPV vaccination had any influence on sexual behavior of vaccinated adolescent girls.

**Methods:**

This was an unmatched comparative study, which used both qualitative and quantitative study methods. It was carried out among 400 primary school girls aged 12 to 15 years in the districts of Nakasongola (vaccinated) and Luwero (non vaccinated). Quantitative data was collected using a questionnaire while qualitative data was obtained using focus group discussions and key informant interviews. The main outcome measure was the number of sexually active girls in each group.

**Results:**

Of the 400 girls, 8 volunteered information that they were sexually active, 5(2.5%) from Luwero (non vaccinated) and 3 (1.5%) from Nakasongola (vaccinated), but there was no statistically significant difference between the 2 groups. HPV vaccination was not significantly associated with being sexually active.

**Conclusion:**

There was no significant difference in sexual behavior between vaccinated and non vaccinated girls.

## Background

Human Papilloma Virus (HPV) particularly types 16 and 18, which are sexually transmitted have been shown to be the cause of cervical cancer [1]. Following establishment of this association, two vaccines have been developed and licensed by the U.S Food and Drug Administration for vaccination against HPV. Vaccination prior to sexual debut, which is about 9 to 13 years of age has been recommended by WHO as a primary prevention strategy that offers greatest protection against HPV infection [2,3]. Vaccination has been ongoing in developed countries. Although the burden of cervical cancer is high in sub Saharan Africa, the uptake of HPV vaccination is still low [4,5]. In 2006, Program for Appropriate Technologies (PATH), an International nonprofit organization that transforms global health through innovation, began demonstration programs in middle and low-resource countries to assess acceptance, feasibility, achievable coverage, and costs associated with HPV vaccination. Projects were conducted in India, Peru, Uganda and Vietnam [6,7]. For Uganda, the demonstration project started in 2008 and 2009 by PATH and Ministry of Health [8]. The vaccine was given to girls in primary five in Ibanda district irrespective of age using a school based approach and girls aged ten years in Nakasongola district during child days plus [8].

There have been concerns that HPV vaccination will result in early sexual indulgence and risky sexual behavior among vaccinated girls because they might think they are protected against a sexually transmitted infection [9,10]. Studies done in developed countries where vaccination started earlier have showed that some respondents thought that HPV vaccination would encourage early sexual indulgence or unsafe sexual behavior, but majority disagreed with that idea and agreed with universal vaccination [11-13]. It is not known if this concern exists in sub-Saharan Africa, Uganda in particular. In spite of these concerns, Bednarczyk et al. in a retrospective cohort study in USA compared clinical markers of sexual activity among HPV vaccinated and non vaccinated 11 to 12 year old girls and found no difference [14]. It has also been shown that preventive methods for early pregnancy for example, do not lead to high risk behaviors. In a study in Seattle, USA by Kirby et al., measures like provision of emergency contraception and increased distribution of condoms in high schools did not result in more sexual encounters, or sexual encounters at younger ages among the students [15,16].

We compared the sexual behavior of HPV vaccinated and non vaccinated adolescent girls in two neighboring districts in Uganda. Luwero, the comparison district, was chosen because it is close to Nakasongola and were thought to have similar social and cultural characteristics. This study was done to provide insight into the unknown surrounding behavior change and presumed increase in sexual activity among the vaccinated girls in Uganda. Specifically, the study was done to compare the sexual behavior of HPV vaccinated and non vaccinated adolescent girls in two neighboring districts in Uganda, and find out if HPV vaccination led to increased sexual activity among adolescent girls.

## Methods

### Participants

We compared the behavior of HPV vaccinated and non-vaccinated primary school girls aged 12 to 15 years in two Ugandan districts, Nakasongola and Luwero. Nakasongola was one of the pilot districts for HPV vaccination campaign initiated in 2008. Luwero was chosen as a comparison district because it is close to Nakasongola, and was thought to have similar socio-demographic and cultural characteristics.

Entry criteria included girls aged 12 to 15 years whose parents gave written consent, and who gave assent to the study. Vaccination status was confirmed by reviewing the vaccination register.

In each district we randomly selected two rural and one urban sub county. Dating was defined as having a special male friend (boyfriend) whom the girl regularly met privately and spent time with. In each sub county, 2 primary schools were randomly selected to participate in the study, for a total of 6 schools in each district. After obtaining permission from the head teacher, girls were provided a written invitation to give to their parents specifying the day of the study. Interested parents came to school with the child on the day of the study, and written informed consent was obtained from each parent, as was assent from each girl. At each school up to 40 girls were recruited. If they were more, we enrolled consecutively until we reached the required number.

Socio-demographic factors including age, class, tribe, religion, presence and level of education of parents and vaccination status of the girls were obtained through self-administered, pretested questionnaire. In addition, a detailed sexual history was obtained from each girl, as well as knowledge and attitudes towards HPV vaccine. Each girl had height and weight measures, and Tanner stage was assessed.

The questionnaires (data collection tools) were pretested before the commencement of the study, in a pilot study done in Wakiso district, which validated their use.

### Statistical analysis

Data were entered into the computer using EPI-INFO version 3.4.1 and analysed using SAS version 12. Our primary outcome was sexual activity, defined as sexual intercourse any time from the time of HPV vaccination (or any time from 10 years of age for unvaccinated girls) to the time of this study. Logistic regression was used in univariate and multivariate analysis to determine the association between sexual activity and HPV vaccination, as well as other potential confounders. Two sided p values ≤ 0.05 was considered statistically significant.

We assumed that 22.6% of enrolled adolescents are sexually active by 15 years of age [17] and we were interested in detecting at least a 30% increase in the number of girls initiating sexual activity after HPV from 22.6% to 29.4%. Therefore, we needed to enroll 196 girls in each district, or 35–40 per school.

### Qualitative study

In each district, 3 schools were randomly selected for focus group discussion. Ten girls, from among those whose parents had come, and were not enrolled in the quantitative study were chosen to participate at each school in a group semi-structured focus group. The discussions were tape recorded. Additionally, five key informant interviews were conducted with the District Health Officer, senior nursing officer and the district inspector of schools in Luwero, and in Nakasongola with the district inspector of schools and the HPV vaccination focal person. All were conducted by the principle investigator. These were done to further get information on the sexual behavior of the girls, and to triangulate the quantitative results. The analysis of qualitative data was done manually after transcription. The recordings that were in Luganda were transcribed directly to English by the research assistant. The ones in English were transcribed in English. The research assistant and principal investigator read through the transcripts and the notes that were taken during the discussions to analyse them. This analysis was done according to the preset themes. Direct quotations from the respondents were used in presentation of study findings.

The study team adhered to the Relevance Appropriateness Transparency Soundness (RATS) guidelines on qualitative research during the conduct of this study.

Ethical approval for the study was obtained from the Department of pediatrics child health, Institutional review board of Makerere University College of Health Sciences, and Uganda National Council of Science and Technology (UNCST) before the study.

## Results

We enrolled 400 girls aged between 12 to 15 years attending primary school classes 5 to 7 in Nakasongola and Luwero districts in Uganda (Table 1). Overall, the mean age of the participants was 12.8 years. The mean age for the vaccinated and non vaccinated girls was 12.5 and 13.1 years respectively. On average, the girls’ weight was 42.7 kg and height was 150.3 cm tall. Most of the girls, 70.5% and 72% from Luwero and Nakasongola respectively were in Tanner stage II and III.

**Table 1:** Baseline Characteristics of the participants

**Parameter**	**Luweero (n = 200)**		**Nakasongola (n = 200)**	
	**Frequency**	**Percent**	**Frequency**	**Percent**
Age (years):				
12 – 13	131	65.5	171	85.5
14 – 15	69	34.5	29	14.5
Tanner stage:				
I	7	3.5	38	19.0
II – III	141	70.5	144	72.0
IV – V	52	26.0	18	9.0
Class:				
P.5	47	23.5	118	59.0
P.6	96	48.0	61	30.5
P.7	57	28.5	21	10.5
Weight (kg):				
**≤** 36	36	18.0	68	34.0
37 – 42	42	21.0	58	29.0
43 – 48	58	29.0	39	19.5
> 48	64	32.0	35	17.5
Height (cm):				
≤ 145.0	41	20.5	56	28.0
145.1 – 150.0	42	21.0	56	28.0
150.1 – 155.0	54	27.0	52	26.0
> 155.0	63	31.5	36	18.0
Living with parents:				
Both parents	91	45.5	124	62.0
Mother	49	24.5	46	23.0
Father	7	3.5	8	4.0
Others	53	26.5	22	11.0
Care taker’s education:				
≤ Primary	68	34.0	56	28.0
> Primary	63	31.5	77	38.5
Religion:				
Catholic	52	26.0	50	25.0
Protestant	49	24.5	80	40.0
Muslim	41	14.2	11	5.5
Born again	51	25.5	43	21.5
Adventist	6	3.0	16	8.0
Others	1	0.5	0	0.0

**Figure 1:**
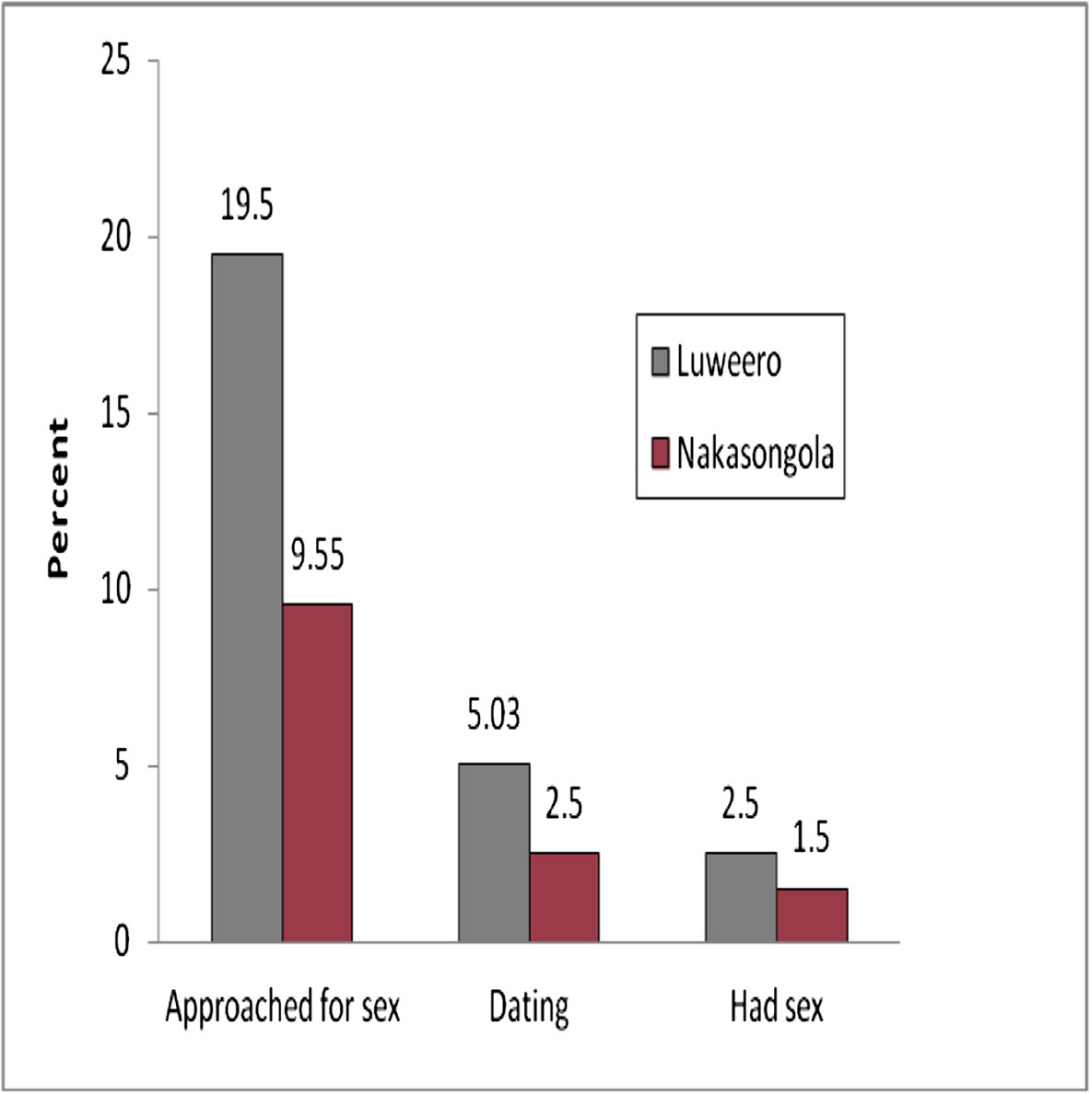
**Proportion of girls involved in three sexual behaviors in each district.** Ten unvaccinated girls (5% from Luwero) were dating and 2.5% (5 girls) reported to have had sex. In Nakasongola, 2.5% (5 girls) were dating.

The unvaccinated girls were more likely to be approached for sex, date or be sexually active compared to the vaccinated girls. This is illustrated in Table 2 and Figure 1. Ten unvaccinated girls (5%) were dating and 5 girls (2.5%) reported to have had sex. In Nakasongola, 5 girls (2.5%) were dating and 3 (1.5%) had had sexual intercourse. No statistical differences were noted in any behavior related to sexual activity between the 2 districts.

**Table 2:** Comparing sexual behaviors between the two districts

**Sexual behavior**		**Nakasongola**	**Luwero**	**OR (95% CI)**	**P – value**
**Approached for sex**	Yes	19	39	0.44 (0.24, 0.78)*	0.0049
	No	181	161		
**Dating**	Yes	5	10	0.48 (0.16, 1.44)	0.2924†
	No	195	190		
**Sexually active**	Yes	3	5	0.59 (0.14, 2.52)	0.7238†
	No	197	195		

*Significant at 5% significance level^†^ C*ells have expected counts less than 5; Fisher’s Exact Test was used*. There was no significant difference in the sexual behavior between the vaccinated and non vaccinated girls. Vaccinated girls were less likely to be approached for sex.

### Factors influencing sexual behavior

**Figure 2:**
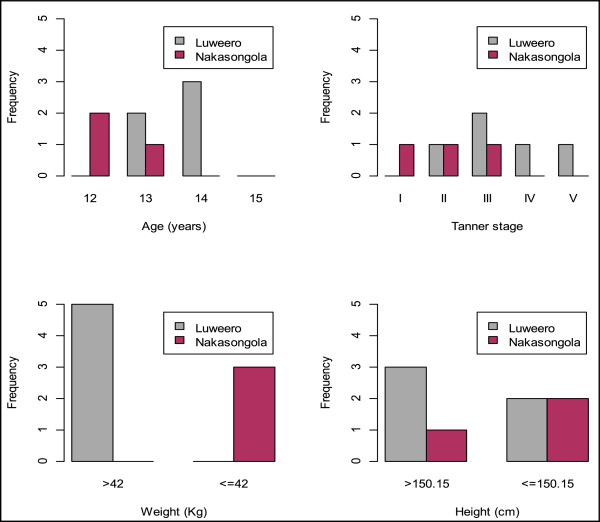
**Bar graphs showing number of girls who were sexually active in each district stratified by age, tanner stage, weight and height.** There was no significant trend of increasing number of sexually active girls with increasing age and tanner stage. Most of the sexually active girls were of higher age, tanner stage, weight and height, and were from Luwero district (non vaccinated) compared to the vaccinated girl.

For the girls who were sexually active, only dating was a statistically significant factor associated with this behavior, OR 127.6 (95% CI 18.3-1356.3, p value < .0001), although the numbers were small. In terms of physical characteristics, most of the sexually active girls were heavier and were unvaccinated, 5 girls were >42 kg and 3 were 42 kg. There were about equal numbers of sexually active girls across the tanner stages, heights and ages assessed. This is illustrated in Figure 2.

### HPV and vaccine knowledge

The proportion of girls who had ever heard about HPV vaccine, knew how HPV is prevented and knew that the vaccine prevents HPV only was statistically significantly higher in Nakasongola than Luwero. Importantly, more girls in Nakasongola did not think that HPV vaccination would make girls become sexually active. A smaller proportion of girls, 6.5% in Nakasongola reported that they had never heard about HPV vaccine compared to 97.5% in Luwero. Most of the Luwero girls reported abstinence and use of condoms as ways of preventing HPV, while most of the Nakasongola girls reported HPV vaccination (Table 3).

**Table 3:** HPV Vaccine Knowledge among study participants

**Parameter**	**Nakasongola (n = 200)**		**Luweero (n = 200)**		**P- value**
	**Freq.**	**Percent (95% CI)**	**Freq.**	**Percent (95% CI)**	
Ever heard about HPV vaccine:					
Yes	187	93.5 (89.1 – 96.5)	5	2.5 (0.8 – 5.7)	*<.0001
No	13	6.5 (3.5 – 10.9)	195	97.5 (94.3 – 99.2)	
Know how HPV is transmitted:					
Yes	83	41.5 (34.6 – 48.7)	79	39.5 (32.7 – 46.6)	0.684
No	117	58.5 (51.3 – 65.4)	121	60.5 (53.4 – 67.3)	
Know how HPV is Prevented:					
Yes	130	65.0 (58.0 – 71.6)	73	36.5 (29.8 – 43.6)	*<.0001
No	70	35.0 (28.4 – 42.0)	127	63.5 (56.4 – 70.2)	
Know that HPV vaccine prevents only HPV:					
Yes	131	65.5 (58.5 – 72.1)	21	10.5 (6.6 – 15.6)	*<.0001
No	69	34.5 (27.9 – 41.5)	179	89.5 (84.4 – 93.4)	
Think that HPV vaccine prevents other STDs:					
Yes	85	42.5 (35.6 – 49.7)	63	31.5 (25.1 – 38.4)	*<.0001
No	105	52.5 (45.3 – 59.6)	82	41.0 (34.1 – 48.2)	
Don’t know	10	5.0 (2.4 – 9.0)	55	27.5 (21.4 – 34.2)	
Think that HPV vaccination will make girls sexually active :					
Yes	14	7.0 (3.9 – 11.5)	18	9.0 (5.4 – 13.9)	<.0001
No	173	86.5 (81.0 – 90.9)	132	66.0 (59.0 – 72.5)	
Don’t know	13	6.5 (3.5 – 10.9)	50	25.0 (19.2 – 31.6)	

*Significant at 5% significance level.

The proportion of girls who had ever heard about HPV vaccine, knew how HPV was prevented and knew that the vaccine prevented HPV only was significantly higher in Nakasongola than Luwero. Significantly, more girls in Nakasongola did not think that HPV vaccination would make girls become sexually active.

### Qualitative study results

Six focus group discussions were held and 52 girls selected from 3 schools in each district for these. A total of five key informant interviews were also held. Data was analyzed in four set themes including; Knowledge of cervical cancer and HPV vaccine, sex education, sexual behavior of girls, and attitude towards influence of HPV vaccination on sexual behavior. This was done to try to triangulate the results from the quantitative study, which relied on self report on sexual behavior.

#### Knowledge about cervical cancer and HPV vaccine

Most of the girls and key informants from Luwero had never heard about cervical cancer or HPV vaccine.

*“I had never heard about HPV vaccine, I am hearing from you”*, said the District Inspector of Schools, Luwero District, but he knew about cancer of the cervix. One girl in one of the schools knew about cervical cancer, because her Aunt had died of cervical cancer, the others did not know.

In comparison to those in Luwero, more girls in Nakasongola and key informants knew about HPV vaccine and cervical cancer. Most of the girls said *“HPV vaccine prevents cervical cancer”.*

### Sexual behavior of girls

#### Engagement in sexual activity

Across both districts, the girls said they were not engaged in sexual activity but knew of others who were, for example one girl said *“our neighbor who was 13 years had sex with a boy and she became pregnant*”, (girl from Nakasongola)*.*

The key informants also reported that young girls do engage in sexual activity. “*Girls used to drop out of school in P5, 6 and 7, some were married off due to pregnancy, but it has reduced over the years because of the intervention by police child protection unit, education department and the probation offices”,* (key informant from Nakasongola). *“Girls in the urban areas generally start sex from as low as 14 years but majority at 18, 17 years. They are seen in antenatal clinic and labour wards. In the rural areas the age is higher, even among those that are well off”,* (Key Informant from Luwero).

#### Reasons for engagement in sexual activity

The main reasons why young girls engage in sexual activity were identified as; peer pressure or joining bad groups, need for good things, being lured by men with gifts or free rides by motor cyclists, lack of parents at home, poverty, feeling grown up, and admiring girls who walk to schools with boys. Again, the reasons reported were similar across both districts. Lack of parents, poverty, and feeling grown up were mostly reported by the rural schools. These reasons were elaborated by some girls as follows; *“some girls need money”* (14 yr old, Nakasongola). “*some think they have grown up”.”some admire their friends’ property and end up going for sex”* (13 year old from Luwero). *‘In most cases they are lured by men with gifts, free lifts by bodabodas”*, (key informant, Luwero). *“In Luwero, poverty used to drive girls to early sex and would get pregnant after the war when people were poor, and there were many orphans but now it is not a major factor.“*(Key informant from Luwero).

#### Influence of HPV vaccination on sexual behavior

Most of the participants in Nakasongola had positive attitude towards HPV vaccination. They thought it would not lead girls to initiate sex early or engage in risky behavior. Some girls thought the vaccine protects against other STDs, but believed that it wouldn’t cause bad sexual behavior. They also thought that the current behavior of girls was not contributed to by HPV vaccination, for example, one girl in Nakasongola said *“A girl will not start sex because she will know the dangers involved.”* (13 year old girl).

A few girls felt that vaccination would increase sexual activity or lead to risky behavior as one said “*some girls don’t listen so even if you immunize them they say I can have sex after all I am immunized.”* (12 year old girl, Nakasongola).

In Nakasongola, two key informants said initially some health workers thought it would lead girls to have sex early. But after being educated, they no longer thought like that.

One key informant also reported that in one school, when the vaccination programme had just started, some parents had fears that it would increase sexual activity among the girls. But after being educated about the program, they changed their thinking. “*Parents were worried that their girls would become sexually active’* (key informant, Nakasongola)*.*

Another key informant said; *some health workers were worried that girls would start sex early because they were taught the reproductive system in detail”.*

Generally, the key informant themselves did not think that vaccination would lead girls to become sexually active early. Participants from Luwero where there was no vaccination had the same thought as one key informant said “*vaccination comes with education, if the girls are taught, they will not misbehave because they will know the dangers*.”

Generally the importance of sex education was emphasized by the key informants, and the girls were less likely to have sex early if they were informed.

## Discussion

We assessed the influence of HPV vaccination on sexual behavior in by comparing sexual behavior of adolescent girls in two neighboring districts in Uganda, about two years following initiation of the vaccination programme. Our study showed that there was no difference in sexual behavior among HPV vaccinated and vaccinated girls. This was shown by the fact that HPV vaccination was not significantly associated with being sexually active. It was also noted that the unvaccinated girls were more likely to date or be sexually active, 2.5% compared to 1.5% although the difference was not statistically significant. In addition, this result could have been dues to chance, because of the small numbers and the wide confidence intervals. We attributed this to their relative physical maturity compared to the vaccinated girls. We did not compare sexual behavior between the rural and urban schools, but Luwero district is closer to the capital city and this could also explain why unvaccinated girls were more likely to date or be sexually active.

In terms of knowledge, 105/200 (52.5%) of girls from Nakasongola and 82/200 (41%) from Luwero thought that the vaccination did not protect against other STDs, but this was not statistically significant. High percentages 173/200 (86.5%) and 132/200 (66%) respectively thought that vaccination would not make girls become sexually active and this was statistically significant (p value <0.0001). With this knowledge it would be expected that the girls would not engage in early sex or risky behavior because they know the consequences of such behavior. Although majority of the girls were of the view that HPV vaccination would not enhance sexual activity, a few of them, 14/200 (7.0%) from Nakasongola and 18/200 (9.0%) from Luwero said that vaccination would make girls start engaging in early sexual activity. Their reason for this was that the girls would think they are protected from STDs. This was also cited in one of the FGDs as one girl said, “*Some girls don’t listen so even if you immunize them they say I can have sex after all I am immunized.”* With these findings, sexual risky behaviors could occur and should be prevented by education and counseling adolescents during vaccination, which was done.

There is paucity of data in Africa regarding influence of HPV vaccination on adolescent sexual behavior. A study with parents of 8–14 year old school girls in England found that 12% of them thought vaccination would lead to early sexual indulgence or unsafe sexual behavior [11]. According to two key informants in our study, parents in one school and some health workers were worried when the vaccination programme had just started that vaccination would make girls become sexually active. One had this to say, “*Parents were worried that their girls would become sexually active”.* However with continued understanding about the vaccine, this belief was no longer there. A recent study in USA compared biomarkers of sexual activity between HPV vaccinated and non vaccinated girls aged 11 to 1 2 years. They looked at contraception counseling, testing or diagnosis of an STD, and pregnancy, and found no difference [14], further discrediting the fears that HPV vaccination leads to sexual promiscuity.

The finding in our study is similar to that of Bednarczyk et al. [14]. Although both studies compared the HPV vaccinated and non vaccinated girls, our study did not have a follow up component and we did not measure clinical markers of sexual activity. In view of the small numbers, we were not able to adequately explore other factors that influence sexual activity among these adolescents. However, this is still the first such study in Sub Saharan Africa, and with these findings, people will be willing to vaccinate their daughters against HPV. In another study with both cross-sectional and longitudinal component in England, Forster AS et al. found that there was no difference in sexual behavior among girls who had and those who had not been offered HPV vaccine. He also found that after a follow up period of six months, the sexual behavior of the vaccinated girls was no different from that of the non vaccinated girls [18]. This study also based on self report of sexual behavior by the adolescents, like in our study. In our study, we interviewed the girls about 2 years following vaccination. Liddon NC et al. in a survey in USA also found no difference in sexual behavior of young women based on vaccination status, although they did not have information on the age at which the respondents got HPV vaccination, to further relate it to their sexual behavior [19].

The strengths of our study included the fact that the qualitative component was included to get more information on sexual behavior of the girls, and the fact that each girl answered her questionnaire in private from the other. Also, different girls from those who answered the questionnaire participated in focus group discussions to avoid bias. Only female research assistants were used to help ensure the girls would feel free and comfortable.

Our study also had some limitations. The study participants were young girls in primary school, who may have feared to answer the questions about personal sexual behavior or give the correct answers. They may have feared to be reported to their parents or teachers, which may have led them to giving us socially desirable responses about sexual their activity. This was minimized by reassurance that the information was confidential and ensuring that each girl answered her questionnaire in private and numbers were used instead of names. Biological markers to confirm previous engagement in sexual activity were not looked for. These could have helped to validate that question; however that was not part of the study. In our study the girls were not matched for age, so most of the girls in Luwero were older and heavier than the Nakasongola group, this could have contributed to the difference in sexual behavior in that there were more sexually active girls un vaccinated girls compared to the vaccinated girls, although the difference was not statistically significant. Studies have shown that older and more physically mature adolescents are more likely to engage in sexual activity [17,20,21].

## Conclusions

There was no difference in sexual behavior between the vaccinated and non vaccinated girls aged 12 to 15 years in Nakasongola and Luwero Districts. From this study, HPV vaccination should be promoted by the stake holders because it does not lead to early sexual debut or risky sexual behavior.

## Competing interests

The authors declare that they have no competing interests.

## Authors’ contributions

JCA, SBK, SK and FM designed the study. JCA collected the data and drafted the first draft of the manuscript. All the authors reviewed and approved the final draft of the manuscript.

## Pre-publication history

The pre-publication history for this paper can be accessed here:

http://www.biomedcentral.com/1471-2458/14/155/prepub
